# Chemical Composition, Antioxidant and Anti-Enzymatic Activities, and In Vitro Insecticidal Potential of *Origanum compactum* (Benth.) Essential Oils

**DOI:** 10.3390/plants13172424

**Published:** 2024-08-30

**Authors:** Mohamed Ouknin, Hassan Alahyane, Jean Costa, Lhou Majidi

**Affiliations:** 1 Regional Center of Agricultural Research of Agadir, National Institute of Agricultural Research (INRA), Avenue Ennasr, BP415 Rabat Principale, Rabat 10090, Morocco; m.ouknin@edu.umi.ac.ma; 2 Laboratory of Nanotechnology, Materials and Environment, Department of Chemistry, Faculty of Science, University Mohammed V, Rabat 10106, Morocco; 3 High Institute of Nursing Professions and Health Techniques, Beni-Mellal 23000, Morocco; alahyanerh@gmail.com; 4 Laboratory of Chemistry of Natural Products, Sciences and Technics Faculty, University of Corsica, 20250 Corse, France; costa_d@univ-corse.fr

**Keywords:** *Origanum compactum*, essential oils, chemical composition, biological activities, contact toxicity, *Ceratitis capitata*

## Abstract

This study aimed to investigate the variation in the chemical composition of *Origanum compactum* essential oils (EOs) from four geographically distinct locations. Additionally, we evaluated their antioxidant properties and potential inhibitory effects on acetylcholinesterase (AChE), tyrosinase, and α-glucosidase enzymes and their insecticidal proprieties. Notably, this research also marks the first examination of the mineral composition of *O. compactum*. The chemical composition was determined using gas chromatography–mass spectrometry (GC-MS), which identified thymol (28.72–80.39%), carvacrol (6.54–61.84%), p-cymene (0.27–8.64%), linalool (1.44–1.96%), and caryophyllene oxide (1.34–1.56%) as the major constituents. Concurrently, inductively coupled plasma atomic emission spectroscopy (ICP-AES) revealed significant levels of macro and microelements, including calcium (295.50–512.20 mg/kg), potassium (195.99–398.45 mg/kg), magnesium (59.70–98.45 mg/kg), and iron (43.55–112.60 mg/kg). The EOs demonstrated notable antiradical activities through DPPH (1,1-diphenyl-2-picrylhydrazyl), FRAP (ferric reducing antioxidant power), and β-carotene bleaching assays. Regarding the insecticidal effect, all studied essential oils showed a significant toxicity against *C. capitata* adults, and the toxicity was dose and time dependent. The highest insecticidal effect was observed for *O. compactum* essential oils collected from Gouman (LC_50_ = 2.515 µL/mL, LC_90_ = 5.502 µL/mL) after 48 h of treatment. Furthermore, at a concentration of 1 mg/mL, the EOs exhibited strong inhibitory effects against AChE (84.75–94.01%), tyrosinase (84.75–94.01%), and α-glucosidase (79.90–87.80%), highlighting their potential as natural inhibitors of these enzymes. The essential oils of *O. compactum* contain components that could be used as a basis for synthetizing derivatives or analogs with potential medicinal applications and pest control properties.

## 1. Introduction

Medicinal plants are rich sources of various secondary metabolites, such as essential oils (EOs), phenolic compounds, and flavonoids, exhibiting diverse biological activities [1,2,3,4]. These compounds are known for their therapeutic properties and have been extensively studied for their potential health benefits and use in crop protection. The Lamiaceae family encompasses several genera that are particularly notable for their medicinal plants. Among these genera, *Origanum* stands out with a diverse range of species. It comprises 38 species, including 6 subspecies and 18 hybrids, highlighting its biodiversity and medicinal potential [5,6].

*Origanum* species play a dual role in culinary and medicinal contexts. In Moroccan traditional medicine, *O. compactum* is highly valued for its diverse therapeutic benefits. Widely used in infusions and decoctions, it effectively treats dysentery, colitis, gastrointestinal disorders, gastric acidity, bronchopulmonary ailments, colds, flus, and various otorhinolaryngeal conditions, including bronchitis [7]. Known for their expectorant, stomachic, stimulating, and tonic properties, *Origanum* species also exhibit antiseptic, antispasmodic, and antitussive qualities, making them indispensable in Moroccan popular medicine. Additionally, *Origanum* plants act as potent disinfectants and contribute aromatic elements to perfumes, highlighting their versatility and cultural significance. Extensive in vitro studies have documented the pharmacological potential of the genus *Origanum*, demonstrating its efficacy against a range of conditions, including antibacterial, anti-inflammatory, antioxidant, antitumor, antifungal, antiviral, and antiparasitic activities [7,8,9].

*Origanum* essential oils (EOs) have been particularly noted for their rich contents of terpenoids and phenolic compounds such as carvacrol, thymol, γ-terpinene, and p-cymene [10,11]. These constituents have demonstrated significant biological activities both in vitro and in vivo, including anticancer, antimicrobial, and antioxidant properties [12,13,14], and insecticidal activities [15,16]. Therefore, the use of natural botanical plant products for pest control has advanced significantly in recent years. In this context, essential oils have been utilized in various formulations for pest control. Several plant species, especially from the Lamiaceae family, contain aromatic compounds with insecticidal properties [17], including against the Mediterranean fruit fly, *C. capitata* [18]. Essential oils extracted from the fresh leaves of *Rosmarinus officinalis* L., *Lavandula angustifolia* Mill., and *Thuja occidentalis* L. have also demonstrated insecticidal effects against *C. capitata* [19].

Besides their pest control properties, plant-derived compounds have demonstrated potential in therapeutic applications. Acetylcholinesterase inhibitors are used to treat Alzheimer’s disease by reducing the breakdown of acetylcholine, which is essential for the central cholinergic system, thereby improving cognitive functions. Some of these effective inhibitors, such as galantamine, are derived from plants, encouraging further research into natural products with potential anti-acetylcholinesterase activity [20,21]. Tyrosinase is crucial for hydroxylating L-tyrosine in melanin production. Abnormal melanin biosynthesis can cause substrate buildup in the skin, leading to hyperpigmentation disorders. While synthetic tyrosinase inhibitors are used as skin whitening agents, they often have significant side effects. Consequently, there is a growing interest in finding safe and effective natural tyrosinase inhibitors [22]. Similarly, the global rise in diabetes has resulted in high annual death rates. This metabolic disorder disrupts blood glucose and serum insulin levels. Researchers are focusing on inhibiting α-glucosidase, an enzyme involved in starch hydrolysis. Inhibiting α-glucosidase helps reduce postprandial blood glucose levels in diabetic patients [23,24].

The objectives of this study were multifaceted. Firstly, we aimed to analyze the variation in chemical compounds present in *O. compactum* collected from four distinct locations, each separated by at least 10 km. Additionally, we assessed the insecticidal activity of the essential oils against *C*. *capitata* adults, as well as their antioxidant activity and inhibitory effects on acetylcholinesterase, tyrosinase, and α-glucosidase enzymes. The overarching goal was to establish correlations between the pharmacological activities exhibited by *O. compactum* oils and their chemical compositions.

## 2. Results

### 2.1. Mineral Composition

The significance of the mineralogical analysis of *O. compactum* stems from the pivotal role minerals play in the biological systems of living organisms. Chemical compounds no longer need to be emphasized to underscore this importance. These minerals, such as Ca, P, Mg, S, K, and Na, intricately contribute to tissue structure and cellular metabolism [25,26,27]. Past research has consistently highlighted the positive impact of mineral intake, including potassium (K), sodium (Na), magnesium (Mg), calcium (Ca), copper (Cu), and zinc (Zn), in mitigating various individual risk factors, notably those associated with cardiovascular disease [28,29,30].

Given the traditional usage of Moroccan oregano in forms like powder, decoction, or infusion, it is imperative to thoroughly investigate its mineralogical composition. Surprisingly, there exists a dearth of prior studies in this realm, thus underscoring the significance of our present study. Consequently, we embarked on determining the mineral composition of the aerial parts of *O. compactum* harvested from four distinct locations.

The mineral analysis of *O. compactum* aerial parts (Table 1), studied for the first time, showed significant variations in the mineral element content, depending on the different harvesting locations.

Calcium stands out as the predominant element in the above-ground sections of *O. compactum* across four distinct harvesting locations, exhibiting concentrations of 512.20 mg/kg (OC1), 220.67 mg/kg (OC2), 325.50 mg/kg (OC3), and 295.50 mg/kg (OC4). This highlights a significant variation in the calcium content among the different locations, suggesting potential influences of environmental factors on nutrient uptake and accumulation in the plant.

The concentration of potassium (K) ranged from 195.99 mg/kg to 398.45 mg/kg (OC3), while the magnesium (Mg) content varied between 59.70 mg/kg (OC4) and 98.45 mg/kg (OC1). Iron (Fe) levels spanned from 43.55 mg/kg (OC4) to 112.60 mg/kg (OC1), and phosphorus (P) concentrations fluctuated from 45.75 mg/kg (OC2) to 69.77 mg/kg (OC1). Additionally, the aluminum (Al) content ranged from 4.86 mg/kg (OC2) to 27.33 mg/kg (OC1) (Table 1).

### 2.2. Chemical Composition of Essential Oils

Table 2 displays the yields of essential oil extracted from *O. compactum* across different locations. The obtained results showed that the yield of extraction varied from 3.45% (Imi ntisslmit) as the lowest average yield to 4.50% (Gouman) as the highest average yield.

**Figure 1:**
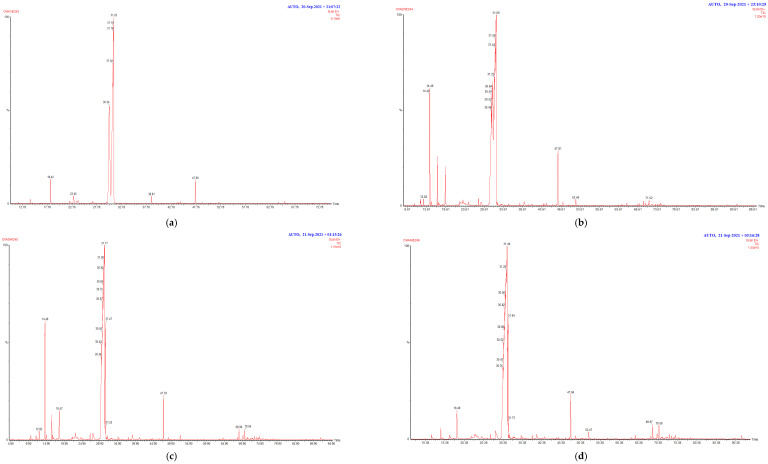
Chromatographic profiles obtained from the GC/MS analysis of O. compactum essential oils collected from four locations: (**a**) Gouman (OC1), (**b**) Tisslmit (OC2), (**c**) Amzray (OC3), and (**d**) Imi ntisslmit (OC4).

The chromatographic analysis of *O. compactum* essential oils allowed the identification of 40 compounds accounting for (94.22–98.27%) of the total chemical composition (Table 2). The *O. compactum* essential oils were dominated by oxygenated monoterpenes (83.42–93.81%), monoterpene hydrocarbons (0.37–93.81%), sesquiterpene hydrocarbons (0.09–2.48%), and oxygenated sesquiterpenes (1.41–1.94%). The main constituents of *O. compactum* essential oils were thymol (28.72–80.39%), carvacrol (6.54–61.84%), p-cymene (0.27–8.64%), linalool (1.44–1.96%), and caryophyllene oxide (1.34–1.56%) (Figure 1).

### 2.3. Toxicity Assessment

The insecticidal activities of the studied EOs are summarized in Table 3. The toxicity of EOs towards adults of *C. capitata* varied over time (*p* < 0.05), showing a time-dependent effect of EO doses. Probit analysis results indicated that the OC1 essential oil exhibited the highest toxicity to *C. capitata* adults (LC_50_ = 2.515 µL/mL, LC_90_ = 5.502 µL/mL) after 48 h of treatment, followed by the OC2 essential oil (LC_50_ = 2.853 µL/mL, LC_90_ = 6.515 µL/mL). In contrast, the OC3 essential oil (LC_50_ = 5.213 µL/mL, LC_90_ = 10.721 µL/mL) and OC4 essential oil (LC_50_ = 7.445 µL/mL, LC_90_ = 16.088 µL/mL) demonstrated significantly lower insecticidal effects against *C. capitata* adults.

### 2.4. Antioxidant Properties

Three distinct methods, namely, the DPPH assay, iron reduction assay (FRAP), and β-carotene bleaching, were employed to evaluate the antioxidant potency of essential oils derived from the aerial parts of *O. compactum.* The sample harvested at Imi ntisslmit showed the most potent antioxidant activity toward DPPH (IC_50_ = 55.96 ± 1.07 µg/mL). The result obtained was less potent than those observed for the synthetic antioxidant gallic acid (IC_50_ = 41.77 ± 0.90 µg/mL) but more potent than BHT (IC_50_ = 185.96 ± 1.24 µg/mL) (Table 4).

In the β-carotene assay, the essential oil derived from *O. compactum* collected at Imi Ntisslmit demonstrated the highest potency, with an IC_50_ value of 30.96 ± 1.27 µg/mL. This indicates a significantly stronger antioxidant activity compared to the pure compounds used as positive controls. Specifically, the IC_50_ value for gallic acid was 63.5 ± 0.91 µg/mL, and for BHT (butylated hydroxytoluene), it was 98.21 ± 0.51 µg/mL. Thus, the *O. compactum* essential oil from Imi ntisslmit exhibited more than twice the antioxidant activity of gallic acid and over three times that of BHT, highlighting its exceptional efficacy in comparison to these standard antioxidants.

The antioxidant capacity of the essential oil, as measured by the ferric reducing antioxidant power (FRAP) assay, is detailed in Table 4. Specifically, the essential oil of *O. compactum* collected from Gouman exhibited significant anti-free radical activity, with an IC_50_ value of 42.50 ± 0.54 µg/mL. Despite this notable activity, the antioxidant effects of the essential oils under study were lower when compared to standard reference substances. Gallic acid and butylated hydroxytoluene (BHT) demonstrated superior antioxidant properties, with IC_50_ values of 10.45 ± 0.25 µg/mL and 36.51 ± 0.60 µg/mL, respectively. This indicates that while the essential oil of *O. compactum* has considerable antioxidant potential, it is less effective than the established reference antioxidants.

### 2.5. Enzyme Inhibitory Effects Activities

The essential oils of *O. compactum*, collected from four different locations, were assessed for their inhibitory effects on acetylcholinesterase, tyrosinase, and α-glucosidase. This in vitro study determined the percentage of inhibition using the Ellman method, with detailed results presented in Table 5. Overall, the inhibitory activity was observed to increase with higher concentrations of the essential oil. Based on the classification by Custódio et al. [31], enzyme inhibition is classified as potent (>50%), moderate (30–50%), low (<30%), or negligible (<5%).

The effectiveness of acetylcholinesterase (AChE) inhibition by the essential oils at the tested doses (0.25, 0.5, 0.75, and 1 mg/mL) was compared to that of the positive control (galanthamine). The results presented in Table 5 demonstrate a positive correlation between the concentration of tested essential oils and their effectiveness. Particularly noteworthy was the remarkable inhibitory efficiency observed for the *O. compactum* essential oil sourced from Gouman, even at a relatively low concentration of 0.75 mg/mL, surpassing the inhibitory efficiency of the positive controls by a notable margin (79.20% compared to 77.30%). The most pronounced inhibitory activity of 94.01% was achieved with the *O. compactum* essential oil from Gouman at a concentration of 1 mg/mL, followed closely by oils from Amzray, Tisslmit, and Imi ntisslmit (93.33%, 85.89%, and 84.75%, respectively, also at 1 mg/mL). This heightened inhibitory effect of *O. compactum* oil is likely attributed to its primary compounds or the synergistic interaction among them.

The inhibitory activity of *O. compactum* essential oils against α-glucosidase is detailed in Table 5. The data indicated that the inhibition efficiency of these essential oils was concentration dependent. Among the samples tested, the essential oil obtained from Tisslmit exhibited the highest inhibition efficiency, achieving an 87.80% inhibition rate at a concentration of 1 mg/mL. This was followed closely by the essential oil from Gouman, which showed an 85.50% inhibition rate at the same concentration. The oils from Imi ntisslmit and Amzray exhibited significant inhibition efficiencies, achieving rates of 80.70% and 79.90% at a concentration of 1 mg/mL, respectively. Furthermore, essential oils from four different localities at the same 1 mg/mL dose showed efficacy just below the positive control (87.33–87.90%). These results suggest that the geographical origin of essential oils may influence their chemical composition/α-glucosidase inhibitory activity.

From Table 5, the tyrosinase inhibitory effect of *O. compactum* essential oils show that the essential oil from Imi ntisslmit exhibited the highest tyrosinase inhibitory activity of 87.40% at 1 mg/mL, followed by those collected from Gouman (83.20%), Tisslmit (83.02%), and Amzray (76.40%) at the same concentration. Additionally, the 1 mg/mL dose of essential oils collected from four localities was very close to the value of the positive control, ranging from 83.44% to 84.80%.

### 2.6. Correlation Analysis

Pearson’s correlation analysis demonstrated a high significant positive correlation between carvacrol and linalool (*r* = 0.981). Additionally, thymol showed a significant negative correlation with both carvacrol (*r* = −0.979) and linalool (*r* = −0.921). Therefore, the correlation findings highlighted that increases in thymol contents were associated with decreases in carvacrol and linalool contents. Conversely, p-cymene exhibited a non-significant correlation with linalool and carvacrol (*r* = −0.441 and *r* = −0.258, respectively). Caryophyllene oxide also displayed a non-significant correlation with thymol and carvacrol (*r* = −0.292 and *r* = 0.302, respectively).

The correlation analysis revealed a moderate negative correlation between carvacrol and antioxidant activities, with Pearson’s coefficients of *r* = −0.647 for DPPH and *r* = −0.549 for β-carotene. Thymol, on the other hand, showed non-significant correlations with DPPH (*r* = 0.489), FRAP (*r* = −0.411), and β-carotene (*r* = 0.376).

The correlation coefficients between the major compounds of the studied EOs (thymol, carvacrol, linalool, and caryophyllene oxide) and their anti-enzymatic activities revealed no significant correlations with AChE, tyrosinase, and α-glucosidase. This lack of significant correlation suggests that other compound classes may be contributing to the observed effects, potentially through synergistic or antagonistic interactions. However, a strong correlation was observed between thymol and the insecticidal effect (*r* = 0.957). For the other major components, only weak to moderate correlations were observed, indicating that the high insecticidal effects of the tested essential oils likely result from the additive toxic effects of multiple compounds.

The correlation analysis between antioxidant and anti-enzymatic activities revealed a strong correlation between FRAP activity and both tyrosinase (*r* = 0.979) and α-glucosidase (*r* = 0.912) activities. However, no significant correlation was found between AChE and antioxidant activities. Additionally, there was no significant correlation between DPPH and tyrosinase (*r* = −0.151) or α-glucosidase (*r* = 0.340) activities, nor between β-carotene and tyrosinase (*r* = −0.137) or α-glucosidase (*r* = 0.343) activities.

## 3. Discussion

The mean yield of *O. compactum* EOs obtained by hydrodistillation varied from 3.45 to 4.50%. Indeed, the extraction yield for all the samples varied from one site to another, which suggests the influence of the habitat region on the yield of essential oil of *O. compactum*. Comparatively, a previous study on *O. compactum* from the Chefchaouen area reported a yield of 2.41% [32], while yields in the Rabat region were notably lower at 1.6% [33] and 2%, as reported by Benazzouz [34]. These variations highlight how the habitat region affects the essential oil yield of *O. compactum*, showing notable differences across various locations. The variances in the extraction yield of essential oils observed among the samples studied could be linked to the distinct soil and geographical characteristics found in the regions and areas where the plant material was collected [35,36,37,38].

The *O. compactum* essential oils were dominated by oxygenated monoterpenes, monoterpene hydrocarbons, sesquiterpene hydrocarbons, and oxygenated sesquiterpenes. The main constituents of *O. compactum* essential oils were thymol, carvacrol, p-cymene, linalool, and caryophyllene oxide. Other studies show that the major compounds of *O. compactum* EOs from the Taounate region of Morocco are thymol (75.53%), carvacrol (18.26%), caryophyllene (1.76%), and methyl linoleate (1.14%) [39]. Variations in the chemical composition of the essential oils studied, as well as those reported in the literature, can be attributed to the soil properties and geographical features of the regions and areas where the plant materials were harvested [11,40,41]. The distinct pedological and environmental conditions in these locations contribute to the diversity in essential oil profiles observed among different studies. The mineralogical analysis using ICP revealed that *O. compactum* is rich in mineral elements, with variations likely influenced by soil fertility and environmental factors, consistent with findings from previous studies on other Lamiaceae species [42,43,44]. Importantly, the levels of these elements remain within the safety guidelines set by the World Health Organization (WHO) [45], confirming the suitability of *O. compactum* for both medicinal and culinary purposes as a food supplement and herbal remedy.

The antioxidant assays highlighted significant activity in all examined essential oils across three methods: DPPH, FRAP, and the β-carotene bleaching test. Studies have identified thymol and carvacrol, prominent constituents of *O. compactum* essential oil, as key contributors to its potent antioxidant properties [46,47]. For instance, El babili et al. [46] reported an IC_50_ value of 2.00 ± 0.10 mg/L for *O. compactum* essential oil in the DPPH assay, attributing this activity to its high carvacrol (36.46%) and thymol (29.74%) contents. Similarly, Quiroga et al. [47] found strong antioxidant activity in *O. compactum* oils from Argentinean species, with IC_50_ values of 0.98 µg/mL and 0.90 µg/mL in the DPPH assay. Additionally, Asensio et al. [48] observed variable ferric reducing antioxidant power (FRAP) values among oregano-type essential oils rich in thymol, highlighting their potent antioxidant effects and potential α-glucosidase inhibition [49,50]. As our correlation analysis indicated a connection between antioxidant activities and carvacrol and no significant correlation with thymol detected in *O. compactum* oils, the findings underscore the robust antioxidant capabilities of these oils, supported by their chemical composition and synergistic interactions among constituents and potential antagonistic activity in the case of thymol.

Our results demonstrated that the lethal concentrations (LC_50_ and LC_90_) of the studied essential oils varied over time post-treatment. At 24 and 48 h after treatment, *O. compactum* OC1 EO showed the highest efficacy against *C. capitata* adults compared to the other tested EOs, with LC_50_ values of 13.979 µL/mL and 2.515 µL/mL, and LC_90_ values of 26.412 µL/mL and 5.502 µL/mL, respectively. The insecticidal effects of the studied EOs are likely due to their chemical compositions, primarily major compounds. These EOs are rich in monoterpenoids, which have been shown to exert significant insecticidal activity against various insect species [18]. Moreover, Lima et al. [51] found that the insecticidal activities of EOs are explained by the effects of their major compounds. On the other hand, research by Benchouikh et al. [52] and Yakhlef et al. [53] indicated that the toxic effect of EOs on insects is not solely due to the main compounds but may also be attributed to the synergistic action of several minor compounds. According to the chemical composition of *O. compactum* EO, it is notably abundant in major compounds known for their insecticidal activities, such as carvacrol, thymol, and *p*-cymene. These compounds have been described as toxic to many insect species by various researchers [54,55,56,57,58]. In a previous study, Alahyane et al. [59] found that the EOs of *Thymus willdenowii*, *Thymus munbyanus*, and *Lavandula maroccana* are rich in carvacrol, thymol, and *p*-cymene, which have acaricidal effects on *Varroa destructor*. In this study, the EOs with carvacrol percentages around 40% and 37% showed LC_50_ values of 2.255 and 2.492 µL/L air, respectively, against *Varroa* mites. In our correlation analysis, thymol was the only compound that showed a strong correlation with insecticidal effects. This finding contrasts with previous studies that have reported insecticidal activities for carvacrol and *p*-cymene as well. These discrepancies suggest that the mechanism of action for EOs likely involves the combined effects of multiple compounds, rather than the influence of a single component.

The study demonstrated that the tested essential oils exhibited significant inhibitory activity against AChE. However, there were no significant correlations between AChE inhibition and the major compounds of the essential oils. This suggests that the observed inhibition is likely due to the combined effects of both major compounds (carvacrol and thymol) and minor constituents, which may interact synergistically to influence the overall activity. The intricate interactions between these components suggest that essential oils may exert their effects on AChE activity through multiple mechanisms [60,61]. In contrast, earlier research by López et al. [62] primarily attributed this inhibition to the main compounds, thymol and carvacrol. This finding opens up a promising avenue for further investigation into the specific mechanisms through which these essential oils exert their inhibitory effects on AChE. The studied EOs are abundant in compounds with hydrophobic regions, likely functioning as competitive inhibitors of the enzyme tyrosinase, which is crucial in melanin biosynthesis. By inhibiting tyrosinase, these compounds can potentially reduce melanin production [63,64]. The anti-free radical properties of essential oils are primarily determined by the structural characteristics of their constituent compounds, with hydroxyl groups playing a crucial role due to their high reactivity [65,66,67].

Several studies have indicated that EOs exhibit multiple modes of action and target various sites in the central nervous system of insects [68,69]. Research on the EO mode of action have focused on AChE inhibition and the blockage of GABA and octopamine receptors [70,71]. In our study, among the four essential oils tested, the OC1 essential oil showed the highest toxic effect. This can be attributed to its high carvacrol content as the main compound compared to the other EOs, as carvacrol was the most active against AChE. Similar results were reported by Lee et al. [70], who found that carvacrol exhibited the strongest AChE inhibitory activity against *Sitophilus oryzae*, followed by thymol and *p*-cymene. Additionally, Park et al. [72] found that thymol and carvacrol had contact toxicities and AChE inhibitory activities. AChE may be an effective target for these two compounds. Recently, carvacrol was reported to bind to the nicotinic acetylcholine receptor (nAChR) in houseflies, suggesting that nAChR could also be a target of carvacrol for its insecticidal activity [73]. In a previous study, the presence of oxygenated groups in sesquiterpenes, particularly ketones or alcohols, was shown to confer an inhibitory capacity for AChE [74]. In fact, Srivastava et al. [75] reported that the inhibitory effect on AChE activity of *E. campaspe* EO was likely due to 1,8-cineole and/or its synergistic effects with minor components such as carvacrol, limonene, α-pinene, and borneol. In this study, the essential oils demonstrated a significant inhibitory capacity for the tyrosinase and α-glucosidase enzymes. This may also explain the insecticidal activity of the studied oils.

## 4. Materials and Methods

### 4.1. Insect Rearing

The larvae of the *C. capitata* species of medflies were collected from infested *Argania spinosa* in the Idaoutanne region, Agadir, Morocco. To obtain adults, these larvae were reared and maintained in our laboratory at the Faculty of Sciences Semlalia, Cadi Ayyad University, Marrakech. The rearing conditions were 25 °C, 60–70% relative humidity (RH), and a natural photoperiod. The medflies were fed a mixture of sugar and solid yeast in a 3:1 ratio, with water provided via cotton imbibed and placed in a plastic cap. All life stages of the medflies were maintained under laboratory conditions (25 ± 1 °C, 75% RH, and a natural photoperiod), as described by Msaad-Guerfali et al. [76]. Adults of both sexes, aged 3 to 4 days after emergence, were used in our bioassays.

### 4.2. Plant Material

*Origanum compactum* Benth.’s aerial components were gathered in Zaouit Ahnsal, Morocco, from four locations spaced at least 10 km apart (Table 6). The taxonomic categorization of the plant materials was conducted following the practical flora of Morocco [77] and submitted to the herbarium at the Faculty of Science and Techniques Errachidia. Subsequently, the harvested plant was air-dried at room temperature.

### 4.3. Plant Mineral Analysis

The aerial parts of *O. compactum* were individually evaluated through a series of meticulous procedures. Firstly, each sample underwent thorough washing with distilled water and subsequent drying in an oven at 80 °C until a consistent weight was achieved. Following this, the dried samples were crushed using a mortar. A 0.5 g portion of the plant material was then subjected to mineralization using a mixture composed of 2 mL of H_2_SO_4_ (98%), 6 mL of HNO_3_ (65%), and 6 mL of H_2_O_2_ (35%). This mixture was heated on a sand bath and boiled for 30 min. After boiling, the resulting suspension was filtered, and the filtrate was adjusted to 25 mL with a 0.1 M nitric acid solution [78]. The metal elements present in the treated solution were identified using a plasma emission spectrometer, specifically the JOBIN-YVON 70 ICP (Inductive Coupled Plasma) ULTIMA AND JY 70.

### 4.4. Essential Oil Isolation and GC-MS Analysis

The essential oil extracted from 100 g of *O. compactum* aerial parts was obtained through a 3 h hydrodistillation process using a Clevenger-type apparatus, following the method outlined in the European Pharmacopoeia [79]. This extraction process was performed in triplicate. Afterward, the water content was removed from the essential oil using anhydrous sodium sulfate, followed by filtration and storage at 4 °C until use and analysis.

For analysis, the obtained essential oil underwent testing using a PerkinElmer Turbo Mass quadrupole detector (Walhton, MA, USA) connected to a Perkin-Elmer 88 Auto system XL. The system was equipped with fused silica capillary columns (60 m × 0.22 mm I.D., film thickness 0.25 μm) with different stationary phases: Rtx-1 (polydimethylsiloxane) and Rtx-wax (polyethylene glycol). Helium was used as the carrier gas at a flow rate of 1 mL/min. The ion source temperature was maintained at 150 °C, and the oven temperature was programmed to increase from 60 °C to 230 °C at a rate of 2 °C/min, followed by an isothermal hold at 230 °C for 35 min. The injector temperature was set at 280 °C, with electron ionization at 70 eV. Mass spectra were recorded over the mass range 35–350 uma, with a split ratio of 1/8 and an injection volume of 0.2 μL of pure oil.

To identify specific compounds, retention indices (IR) were calculated using both non-polar and polar columns, and comparisons were made with authentic compounds, literature data, or through computer matching of mass spectra with those in commercial or internal libraries. The internal library comprised data from authentic compounds documented in the literature [80].

### 4.5. Contact Toxicity Assessment

The topical bioassay was conducted as outlined by Ghalbane et al. [18]. Five flies of both sexes were randomly chosen and chilled at 6 °C for 5 min. Using fine forceps, the immobilized flies were individually handled, and 1 µL of the test solution was applied to the dorsum of each fly using a hand-held micro-applicator. The concentrations tested were 20, 10, 5, and 2.5 µL/mL. All concentrations were prepared from an initial concentration (100 µL/mL) of essential oils using ethanol. Ethanol was used as the control. The flies were provided with a dry diet (a mixture of sugar and solid yeast in a 3:1 ratio) and water. A total of five repetitions were conducted for each concentration, and the mortality was recorded daily for two days.

### 4.6. Antioxidant Activities

#### 4.6.1. DPPH Assay

The effectiveness of *O. compactum* essential oils against radicals was evaluated using the DPPH (2,2-diphenyl-1-picrylhydrazyl) free radical scavenging assay, following the protocol described by Ouknin et al. [38]. In summary, different dilutions of the essential oils and their key active components were mixed with a 0.4 mM DPPH methanolic solution in a ratio of 50 μL to 5 mL. After a 30 min incubation in the darkness, the absorbance of the mixture was measured at 517 nm using a Uviling 9400 (SECOMAM) spectrophotometer. Butylated hydroxytoluene (BHT) and gallic acid were employed as positive controls. The radical scavenging activity was determined using Formula (1):

(1) $$DPPH\;Scavenging\;effect\left(\%\right)=\left(\frac{A_{0}-A_{1}}{A_{0}}\right)\times 100$$

A_0_ denotes the absorbance of the control after a 30 min period, while A_1_ represents the absorbance of the sample after 30 min.

#### 4.6.2. Reducing Power Determination (FRAP)

The assessment of Fe^3+^ reduction by *O. compactum* essential oils was performed following the method outlined by Oyaizu [81]. BHT and gallic acid were used as reference compounds. The experiment was replicated three times, and the IC_50_ values were reported as means ± standard deviations (SDs).

#### 4.6.3. β-Carotene Bleaching Test

The antioxidant capacity of *O. compactum* essential oil was evaluated by measuring its ability to inhibit the bleaching of β-carotene in a linoleic acid system using a method modified from Koleva et al. [82]. Gallic acid and BHT were employed as positive controls, while pure ethanol replaced the essential oil for the negative control. The antioxidant activity of *O. compactum* was quantified based on the degree of β-carotene bleaching using Equation (2):

(2) $$I\%=\left(\frac{A_{\beta -carotene\;after\;2h}}{A_{initial\beta -carotene}}\right)\times 100\;$$

where A_β-carotene after 2h_ represents the absorbance values of β-carotene remaining in the samples after a 2 h assay, and A_initial β-carotene_ is the absorbance value of β-carotene at the beginning of the experiment. All tests were carried out in triplicate, and the oil concentration providing 50% inhibition (IC_50_) was obtained by plotting the inhibition percentage versus the concentration of oil used.

### 4.7. Enzyme Inhibitory Activities

#### 4.7.1. AChE Inhibition

Cholinesterase inhibitory activity was evaluated using a microplate reader following the method outlined by Ellman et al. [83], with minor adjustments. The assay commenced by combining 25 μL of 15 mM ATCI with 125 μL of 3 mM DTNB. Subsequently, 50 μL of 100 mM phosphate buffer (pH 8.0), 25 μL of essential oil concentrations (0.25, 0.5, 0.75, or 1 mg/mL), and either galanthamine at 25 μg/mL or buffer as positive and negative controls, respectively, were added. Lastly, 0.28 U/mL AChE was introduced before measuring the absorbance at 405 nm over 5 min. A control with buffer instead of the plant essential oil was included to determine the percentage of AChE inhibition. The experiment was conducted in triplicate.

#### 4.7.2. Tyrosinase Inhibition

Spectrophotometry was employed to assess tyrosinase inhibitory activity following the procedure outlined by Masuda et al. [84]. The assay commenced with the addition of either *O. compactum* essential oil at concentrations of 0.25, 0.5, 0.75, or 1 mg/mL, or phosphate buffer as a blank, mixed with 80 μL of phosphate buffer (pH 6.8). Subsequently, 40 μL of L-DOPA and 40 μL of tyrosinase were introduced. Kojic acid at a concentration of 200 μg/mL served as the reference standard. Absorbance readings were taken at 475 nm, and inhibitory activity was calculated as a percentage. The experiment was conducted in triplicate.

#### 4.7.3. α-Glucosidase Inhibition

The assessment of α-glucosidase inhibition followed the protocol outlined by Kwon et al. [85]. Various concentrations (0.25, 0.5, 0.75, and 1 mg/mL) of *O. compactum* essential oil or acarbose (used as a positive control) at 1 mg/mL were mixed with 50 μL of phosphate buffer (0.1 M, pH 6.9) containing yeast α-glucosidase (1.0 U/mL). The mixture was then incubated at room temperature for 10 min, after which 50 μL of a p-nitrophenyl-α-D-glucopyranoside solution (5 mM) was added. Following another 10 min incubation, the absorbance was measured at 405 nm to determine the α-glucosidase inhibitory activity as a percentage. Each experiment was conducted in triplicate.

### 4.8. Statistical Analysis

SPSS 25.0 statistical software facilitated the statistical analysis, encompassing a descriptive data analysis comprising means and standard deviations (SDs). Additionally, Tukey’s test (*p* ≤ 0.05) was employed to compare the means across all concentrations and individual enzymes. The lethal concentrations (LC_50_ and LC_90_ values) were estimated using probit analysis [86]. The Chi-square and confidence intervals (95%) were also estimated using SPSS 25.0 statistical software. The correlation analysis between the chemical composition and biological activities was analyzed using Pearson’s coefficients, calculated with SPSS 25.0.

## 5. Conclusions

The results of this study indicate that the chemical profile of *O. compactum* essential oils is influenced by the geographical location. The GC-MS analysis identified the primary constituents as thymol, carvacrol, and p-cymene. Our findings suggest that the higher antioxidant potential of Moroccan oregano oils can be attributed to their elevated thymol and/or carvacrol contents. Additionally, the studied plants are rich in essential minerals such as potassium (K), calcium (Ca), magnesium (Mg), phosphorus (P), and iron (Fe). These minerals can contribute to dietary nutrition, offering a healthier alternative to other potentially toxic sources. This study proposes that the essential oils from these oregano species could serve as natural antioxidants and food preservatives. Furthermore, the essential oils exhibited inhibitory effects on the AChE, tyrosinase, and α-glucosidase. This suggests potential therapeutic applications for these oils in managing Alzheimer’s disease, skin disorders, and type 2 diabetes mellitus. *O. compactum* essential oils are environmentally friendly and biodegradable products. OC1 and OC2 EOs appear to have potential as effective alternatives for controlling Mediterranean fruit fly. However, further studies are necessary on their effects on human and non-target organisms, including pollinators and honeybees. These *O. compactum* oils have shown the strongest toxicity in contact application, but their low aqueous solubility high and volatility can reduce their efficacy in semi-field and field conditions. To overcome these issues, the encapsulation of EOs in nanoparticles is recommended. Several studies have evaluated the effectiveness of EOs encapsulated in different nanoparticles [87,88] and found that encapsulation improved their stability and provided sustained release, enhancing their biological effects [88]. This alternative strategy could be an effective tool for integrated *C. capitata* management under field conditions.

## Figures and Tables

**Table 1:** Descriptive statistics for the contents of heavy metals and oligo-elements *mg/kg* (mkg^−1^) in *O. compactum*.

	OC1		OC2		OC3		OC4	
	Aerial Part		Aerial Part		Aerial Part		Aerial Part	
	Mean	S.D	Mean	S.D	Mean	S.D	Mean	S.D
Al	27.33	1.49	4.86	0.2	19.45	2.45	22.30	1.64
As	0.03	0.01	0.04	0.01	0.07	0.02	0.03	0.01
B	0.25	0.01	0.28	0.04	0.62	0.08	0.67	0.01
Ba	0.19	0.02	0.39	0.11	0.82	0.19	0.69	0.01
Ca	512.20	5.50	220.67	3.25	325.50	10.20	295.50	8.44
Cr	0.11	0.01	0.20	0.12	0.67	0.15	0.06	0.01
Cu	0.23	0.01	0.08	0.03	0.25	0.07	0.03	0.01
Fe	112.60	2.20	79.50	1.80	59.70	5.40	43.55	1.22
K	213.45	9.50	195.99	13.60	398.45	11.50	322.25	10.50
Mg	98.45	2.20	65.50	4.20	77.60	6.37	59.70	3.50
Mn	0.50	0.02	0.33	0.1	1.01	0.09	1.65	0.10
Na	11.45	0.08	9.30	0.70	7.60	0.3	16.32	0.15
Ni	0.21	0.03	0.15	0.07	0.92	0.04	0.22	0.01
P	69.77	1.46	45.75	4.10	66.90	2.50	56.45	1.50
Pb	0.01	0.01	0.21	0.01	0.25	0.03	0.06	0.01
Si	0.13	0.03	3.95	0.33	2.79	0.25	1.13	0.01
Sr	0.16	0.02	0.30	0.02	0.79	0.04	0.7	0.01
Zn	0.10	0.03	0.39	0.05	0.55	0.02	0.63	0.01

*Origanum compactum* samples collected from Gouman (OC1), Tisslmit (OC2), Amzray (OC3), and Imi ntisslmit (OC4).

**Table 2:** Chemical profiling (GC-MS) of *O. compactum* essential oils harvested in four locations.

No. ^a^	Compound Name	RI l ^b^	RIa ^c^	OC1 ^d^	OC2 ^d^	OC3 ^d^	OC4 ^d^
1	methyl isovalerate	721	720	_	_	0.02	_
2	2-hexenal	830	825	_	0.02	0.03	_
3	(Z)-3-hexen-1-ol	831	835	_	0.01	0.01	0.19
4	3-heptanone	865	863	_	0.01	0.01	_
5	α-thujene	932	924	_	0.03	0.06	_
6	α-pinene	936	933	_	0.08	0.17	_
7	camphene	950	947	_	0.02	0.03	_
8	1-octen-3-ol	963	961	0.11	0.31	0.23	0.19
9	3-octanone	964	965	0.04	0.18	0.12	0.09
10	β-pinene	978	974	_	0.03	0.05	
11	3-octanol	981	980	_	_	_	0.01
12	β-myrcene	987	982	0.01	0.29	0.34	0.02
13	δ-3-carene	1010	1008	_	0.02	0.03	_
14	α-terpinene	1013	1011	_	0.01	0.02	0.01
15	*p*-cymene	1015	1014	0.27	7.23	8.64	0.34
16	eucalyptol	1024	1023	0.05	_	_	0.05
17	limonene	1025	1024	_	0.14	0.16	_
18	γ-terpinene	1051	1050	0.04	1.54	0.75	0.02
19	trans-sabinene hydrate	1051	1055	0.05	0.19	0.3	0.28
20	cis-linalol oxide	1072	1059	0.04	0.09	0.11	0.04
21	1-nonen-3-ol	1058	1063	0.02	0.03	0.04	_
22	fenchone	1076	1072	0.01	_	_	_
23	trans-linalol oxide	1072	1074	0.07	_	_	_
24	meta-cymenene	1073	1075	_	0.16	0.14	_
25	cis-sabinene hydrate	1083	1081	_	0.01	_	_
26	linalool	1086	1085	1.96	1.78	1.44	1.54
27	cis-p-mentha-2-en-1-ol	1108	1110	_	0.01	0.01	_
28	verbenol	1139	1132	_	0.03	0.02	_
29	endo-borneol	1150	1153	0.33	_	_	_
30	terpinen-4-ol	1164	1165	1.1	0.52	0.16	0.17
31	α-terpineol	1179	1176	0.66	0.42	0.14	0.23
32	trans-piperitol	1192	1195	0.01	_	_	_
33	pulegone	1213	1221	0.3	0.33	0.76	1.07
34	carvacrol methyl ether	1226	1227	0.08	0.19	0.17	0.13
35	thymol	1267	1273	28.72	31.89	72.89	80.39
36	carvacrol	1278	1285	61.84	47.37	6.54	7.44
37	piperitenone	1315	1317	0.02	0.08	0.1	0.11
38	eugenol	1331	1331	0.06	0.02	0.02	0.02
39	caryophyllene	1421	1420	0.5	_	_	_
40	aromadendrene	1443	1447	0.02	0.02	_	_
41	α-humulene	1456	1452	0.04	_	_	_
42	γ-muurolene	1471	1472	0.01	0.02	0.01	0.01
43	α-muurolene	1496	1492	0.02	0.01	0.01	_
44	β-bisabolene	1483	1501	0.06	0.04	0.01	0.01
45	γ-cadinene	1507	1508	0.07	0.04	0.02	0.03
46	calamenene	1517	1511	0.03	0.01	0.01	0.01
47	δ-cadiene	1520	1516	0.12	0.06	0.03	0.04
48	spathulenol	1572	1566	0.05	0.02	_	0.04
49	caryophyllene oxide	1578	1572	1.56	1.82	1.34	1.74
50	humulene epoxide II	1601	1597	_	0.10	0.07	_
Monoterpene hydrocarbons				0.37	9.39	10.08	0.44
Oxygenated monoterpenes				93.81	83.60	83.42	91.91
Sesquiterpene hydrocarbons				2.48	0.20	0.09	0.09
Oxygenated sesquiterpenes				1.61	1.94	1.41	1.78
Total identified compounds				98.27	95.13	95.00	94.22

^a^ The order of elution on an apolar column is given (Rtx-1). ^b^ Retention indices on an apolar column reported in the literature; ^c^ retention indices on the apolar column (Rtx-1). ^d^ Relative percentages of components (%) are calculated for GC peak areas on the apolar column (Rtx-1), except for components with identical RIa (concentrations on the polar column are given); _: not detected.

**Table 3:** Insecticidal activities (LC_50_ and LC_90_) of essential oils against *C. capitata* adults after 24 and 48 h of treatment.

Essential Oil *	T(h)	LC_50_ (µL/mL) (95% LD)	LC_90_ (µL/mL) (95% LD)	χ^2^	DF
OC1	24	13.979 (9.929–23.891)	26.412 (17.814–47.183)	12.667	23
	48	2.515 (1.846–3.202)	5.502 (4.187–7.324)	11.164	23
OC2	24	21.559 (13.971–44.689)	61.227 (32.668–190.732)	18.853	23
	48	2.853 (2.022–3.738)	6.515 (4.879–9.084)	11.487	23
OC3	24	58.907 (30.391–204.549)	104.979 (49.68–431.667)	9.786	23
	48	5.213 (3.778–7.256)	10.721 (7.837–16.831)	10.981	23
OC4	24	91.29 (42.355–407.43)	219.189 (79.387–1901.429)	11.674	23
	48	7.445 (5.407–11.164)	16.088 (10.712–32.996)	13.481	23

* *O. compactum* essential oils collected from Gouman (OC1), Tisslmit (OC2), Amzray (OC3), and Imi ntisslmit (OC4).

**Table 4:** Antioxidant activity (IC_50_ µg/mL) of the studied essentials oils.

	DPPH (IC_50_ µg/mL)	FRAP (IC_50_ µg/mL)	β-Carotene (IC_50_ µg/mL)
OC1	55.96 ± 1.07 ^b^	105.78 ± 2.14 ^e^	30.96 ± 1.27 ^a^
OC2	109.15 ± 2.01 ^d^	59.69 ± 2.06 ^c^	83.91 ± 1.30 ^d^
OC3	158.54 ± 4.50 ^e^	92.48 ± 1.77 ^d^	121.03 ± 0.70 ^f^
OC4	87.89 ± 1.05 ^c^	42.50 ± 0.54 ^b^	52.74 ± 1.16 ^b^
Gallic acid	41.77 ± 0.90 ^a^	10.45 ± 0.25 ^a^	98.21 ± 0.51 ^e^
BHT	185.96 ± 1.24 ^f^	36.51 ± 0.60 ^b^	75.15 ± 1.33 ^c^

Values are expressed as means ± SEs (*n* = 3). In the same column, values marked with different letters indicate significant differences (*p* < 0.05).

**Table 5:** Enzyme inhibitory activity: AChE, tyrosinase, and α-glucosidase.

Plants Code	Essential Oil Doses (mg/mL)	AChE (%)	Tyrosinase (%)	α-Glucosidase (%)
OC1	0.25	45.00 ± 0.77 ^a^	42.45 ± 0.43 ^a^	47.66 ± 0.12 ^a^
	0.5	61.12 ± 0.53 ^b^	57.66 ± 0.70 ^b^	57.50 ± 0.50 ^b^
	0.75	79.20 ± 0.50 ^c^	70.45 ± 0.30 ^c^	67.20 ± 0.60 ^c^
	1	94.01 ± 0.70 ^d^	83.20 ± 0.60 ^d^	85.50 ± 0.30 ^d^
	Positive control	77.30 ± 0.40 ^c^	83.44 ± 0.73 ^e^	87.33 ± 0.50 ^d^
OC2	0.25	52.45 ± 0.44 ^a^	53.12 ± 0.23 ^a^	54.11 ± 0.40 ^a^
	0.5	67.13 ± 0.50 ^b^	66.79 ± 0.50 ^b^	59.20 ± 0.33 ^b^
	0.75	78.40 ± 0.90 ^c^	76.11 ± 0.20 ^c^	68.48 ± 0.40 ^c^
	1	85.89 ± 0.40 ^d^	83.02 ± 0.10 ^d^	87.80 ± 0.60 ^d^
	Positive control	78.70 ± 0.50 ^c^	84.80 ± 0.33 ^d^	87.50 ± 0.23 ^d^
OC3	0.25	54.60 ± 0.70 ^a^	48.75 ± 0.70 ^a^	44.20 ± 0.40 ^a^
	0.5	70.10 ± 0.20 ^b^	57.90 ± 0.50 ^b^	57.30 ± 0.13 ^b^
	0.75	84.33 ± 0.90 ^d^	66.10 ± 0.55 ^c^	65.10 ± 0.05 ^c^
	1	93.33 ± 0.50 ^d^	76.40 ± 0.66 ^d^	79.90 ± 0.22 ^d^
	Positive control	78.23 ± 0.43 ^c^	84.40 ± 0.30 ^e^	87.90 ± 0.08 ^e^
OC4	0.25	41.80 ± 0.65 ^a^	54.80 ± 0.40 ^a^	52.50 ± 0.80 ^a^
	0.5	51.33 ± 0.44 ^b^	68.30 ± 0.54 ^b^	59.80 ± 0.20 ^b^
	0.75	68.11 ± 0.67 ^c^	77.70 ± 0.43 ^c^	69.53 ± 0.50 ^c^
	1	84.75 ± 0.66 ^e^	87.40 ± 0.60 ^d^	80.70 ± 0.70 ^d^
	Positive control	78.89 ± 0.67 ^d^	84.70 ± 0.23 ^d^	87.80 ± 0.30 ^e^

Values are expressed as means ± SEs (*n* = 3). The same uppercase letters in the same column and the same lowercase letters in the same row show that there is no significant difference in AChE, tyrosinase, α-glucosidase activities and the applied essential oil doses, respectively (Tukey’s test; *p* < 0.05).

**Table 6:** Harvesting sites, collecting times, and essential oil yields of *O. compactum* species.

Plant Species	Species Code	Local Name	Harvesting Site	Collection Time	Voucher Specimen	GPS Coordinates	Oil Yield (% (w/w))
*Origanum compactum* Benth. (OC)	OC1	Izzikni	Gouman	June 2021	ER-22-10	31°49′43.9″ N, 6°06′16.4″ W	4.50 ± 0.15
	OC2	Izzikni	Tisslmit	June 2021	ER-22-11	31°49′31.4″ N, 6°06′22.2″ W	3.90 ± 0.20
	OC3	Izzikni	Amzray	June 2021	ER-22-12	31°50′01.7″ N, 6°07′27.9″ W	4.20 ± 0.30
	OC4	Izzikni	Imi ntisslmit	June 2021	ER-20-13	31°50′18.3″ N, 6°06′07.7″ W	3.45 ± 0.10

## Data Availability

The raw data are available from the corresponding author (Lhou Majidi) upon reasonable request.
